# Identification of Ferroptosis-Related Genes in Alzheimer’s Disease Based on Bioinformatic Analysis

**DOI:** 10.3389/fnins.2022.823741

**Published:** 2022-02-07

**Authors:** Ying Wang, Guohua Chen, Wei Shao

**Affiliations:** Department of Neurology, Wuhan Hospital of Traditional Chinese and Western Medicine, Tongji Medical College, Huazhong University of Science and Technology, Wuhan, China

**Keywords:** Alzheimer’s disease (AD), WGCNA, ferroptosis, apoptosis, GEO

## Abstract

**Introduction:**

Alzheimer’s disease (AD) is the most prevalent cause of dementia, and emerging evidence suggests that ferroptosis is involved in the pathological process of AD.

**Materials and Methods:**

Three microarray datasets (GSE122063, GSE37263, and GSE140829) about AD were collected from the GEO database. AD-related module genes were identified through a weighted gene co-expression network analysis (WGCNA). The ferroptosis-related genes were extracted from FerrDb. The apoptosis-related genes were downloaded from UniProt as a control to show the specificity of ferroptosis. The overlap was performed to obtain the module genes associated with ferroptosis and apoptosis. Then the Gene Ontology (GO) and Kyoto Encyclopedia of Genes and Genomes (KEGG) pathway enrichment analyses and the protein-protein interaction (PPI) were conducted. Cytoscape with CytoHubba was used to identify the hub genes, and the Logistic regression was performed to distinguish the AD patients from controls.

**Results:**

53 ferroptosis-related module genes were obtained. The GO analysis revealed that response to oxidative stress and starvation, and multicellular organismal homeostasis were the most highly enriched terms. The KEGG analysis showed that these overlapped genes were enriched not only in renal cell carcinoma pathways and central carbon metabolism in cancer, but also in autophagy-related pathways and ferroptosis. Ferroptosis-related hub genes in AD (JUN, SLC2A1, TFRC, ALB, and NFE2L2) were finally identified, which could distinguish AD patients from controls (P < 0.05). The area under the ROC curve (AUC) was 0.643. Apoptosis-related hub genes in AD (STAT1, MCL1, and BCL2L11) were also identified and also could distinguish AD patients from controls (P < 0.05). The AUC was 0.608, which was less than the former AUC value, suggesting that ferroptosis was more special than apoptosis in AD.

**Conclusion:**

We identified five hub genes (JUN, SLC2A1, TFRC, ALB, and NFE2L2) that are closely associated with ferroptosis in AD and can differentiate AD patients from controls. Three hub genes of apoptosis-related genes in AD (STAT1, MCL1, and BCL2L11) were also identified as a control to show the specificity of ferroptosis. JUN, SLC2A1, TFRC, ALB, and NFE2L2 are thus potential ferroptosis-related biomarkers for disease diagnosis and therapeutic monitoring.

## Introduction

Alzheimer’s disease (AD) is the most prevalent cause of dementia, accounting for approximately 60–80% of all cases (Gbd 2016 Dementia Collaborators, 2019). The exact pathogenesis of AD is still not fully elucidated (Zhang et al., 2021). Ferroptosis is an iron-dependent lipid peroxidation-driven cell death, and emerging evidence suggests that it is involved in the pathological process of AD (Lane et al., 2018; Weiland et al., 2019). In addition, several characteristics of the pathogenesis of AD were consistent with those of ferroptosis, such as excess iron accumulation, elevated lipid peroxides (Zhang et al., 2012; Hambright et al., 2017; Ayton et al., 2019). Therefore, ferroptosis is increasingly being recognized as a unique cell death mechanism participating in the pathogenesis of AD. However, more direct evidence is needed to be presented (Chen et al., 2021). Apoptosis is the spontaneous and orderly death of cells, which involves the activation, expression and regulation of a series of genes, and it is a biological process that plays an essential role in normal physiology (Obulesu and Lakshmi, 2014). It is now generally accepted that massive neuronal death due to apoptosis is a common characteristic in the brains of patients suffering from neurodegenerative diseases, and apoptotic cell death has been found in neurons and glial cells in AD (Shimohama, 2000; Sharma et al., 2021).

Current studies on ferroptosis and AD are mainly focused on two aspects: one is the mechanism of ferroptosis in the pathological process of AD, mainly discussing how ferroptosis participates in the AD (Masaldan et al., 2019; Jakaria et al., 2021); the second is the clinical efficacy study of ferroptosis inhibitors in AD, mainly to explore whether ferroptosis as a drug target of AD can effectively delay the progression of AD (Yan and Zhang, 2019; Plascencia-Villa and Perry, 2021; Vitalakumar et al., 2021). The purpose of this study is to investigate the association between ferroptosis-related genes and AD with the gene level, which is a supplement to existing studies and also a reference for ferroptosis as a therapeutic target for AD. These hub genes identified by this study could also serve as the ferroptosis-related biomarkers for disease diagnosis and therapeutic monitoring.

## Materials and Methods

### Microarray Data Processing

**FIGURE 1:**
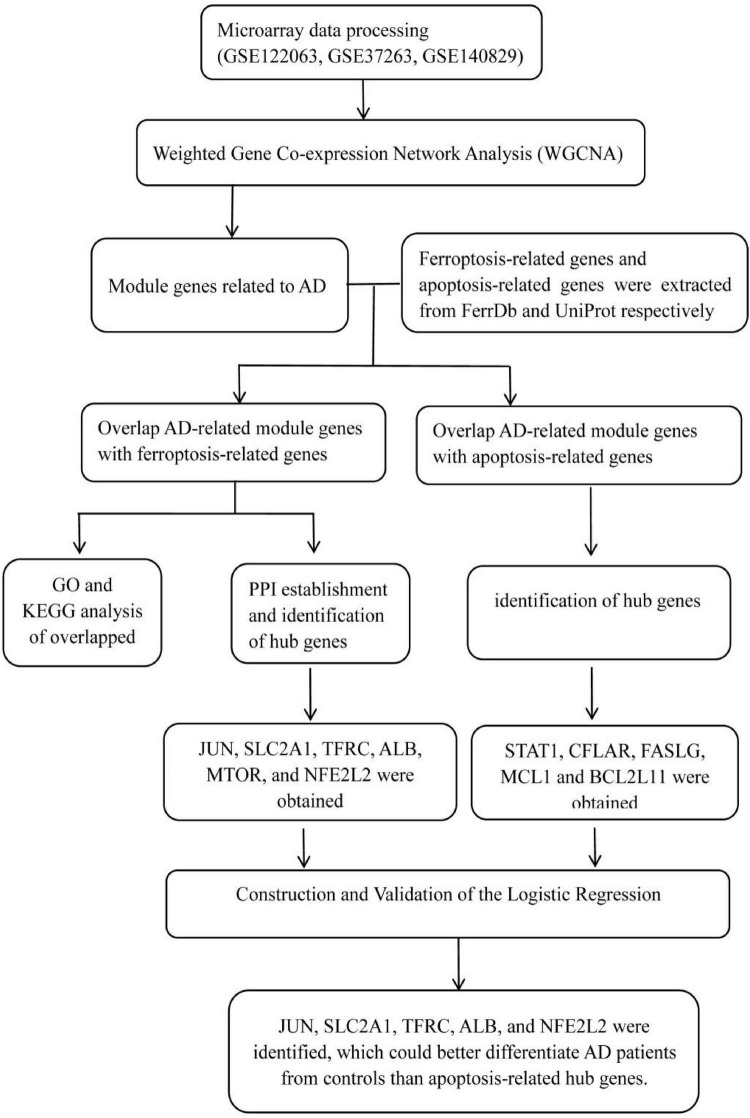
The workflow chart of data preparation, processing, analysis, and validation.

Three microarray datasets (GSE122063, GSE37263, and GSE140829) of AD were collected from the GEO database^1^. GSE122063 was based on the platforms of the GPL16699 (Mckay et al., 2019); GSE37263 was based on the platforms of the GPL5175 (Tan et al., 2010); and GSE140829 was based on the platforms of the GPL15988. Data for 56 AD patients and 44 control samples from GSE122063, 8 AD patients and 8 control samples from GSE37263, and 182 AD patients and 207 control samples from GSE140829 were analyzed in our study. A flow diagram of the study is shown in Figure 1.

### Weighted Gene Co-expression Network Analysis

Firstly, the expression profiles of three datasets were removed from the batch effect for further analysis. The gene co-expression network was constructed with an R package termed “weighted gene co-expression network analysis (WGCNA)” (Langfelder and Horvath, 2008, 2012). The Adjacency matrix was constructed by a weighted correlation coefficient. Subsequently, the adjacency matrix was transformed into a topological overlap matrix (TOM). Then, hierarchical clustering was performed to identify modules, and the eigengene was calculated. Finally, we assessed the correlation between phenotype (i.e., AD or control samples) and each module by Pearson’s correlation analysis and identified AD-related modules. The genes in these modules were considered as AD-related module genes.

### The Extraction of Ferroptosis-Related Genes From FerrDb and Apoptosis-Related Genes From UniProt

FerrDb^2^ is an artificial ferroptosis database for the management and identification of ferroptosis-related markers and regulatory factors, as well as ferroptosis-related diseases (Zhou and Bao, 2020). Therefore, ferroptosis-related genes were downloaded from this database for further analysis. The UniProt Knowledgebase is the central hub for the collection of functional information on proteins, with accurate, consistent and rich annotation, and thus apoptosis-related genes were extracted from UniProt^3^.

### Overlap Alzheimer’s Disease-Related Module Genes With Ferroptosis-Related Genes and Apoptosis-Related Genes, Respectively

Ferroptosis-related genes were downloaded from FerrDb and apoptosis-related genes were downloaded from UniProt. We overlapped these genes with AD-related module genes derived from WGCNA, respectively. The Venn diagram was used to describe the details of the overlapped genes.

### Gene Ontology and Kyoto Encyclopedia of Genes and Genomes Enrichment Analysis of Overlapped Genes

Functional enrichment analysis was performed in three domains of GO, including biological process (BP), cellular component (CC), and molecular function (MF). The KEGG database contains datasets of pathways involving biological functions, diseases, chemicals, and drugs. The enrichment analysis was carried out by clusterProfiler R package to determine the biological functions of the genes and associated pathways (Yu et al., 2012).

### Protein-Protein Interaction Establishment and Identification of Hub Genes

An online tool (Search Tools for the Retrieval of Interacting Genes, STRING^4^) was used to analyze protein interactions. The PPI pairs were screened by confidence score (>0.40), and the PPI network was visualized by the Cytoscape V3.9.0 software (Shannon et al., 2003). Three indicators (Degree, closeness, and Betweenness) were calculated through CytoHubba to evaluate the importance of each node, and the top 10 nodes were selected. The hub genes were their common nodes.

### Construction and Validation of the Logistic Regression

To effectively differentiate the AD patients from controls, the logistic regression was constructed, and to evaluate the performance of the logistic regression model for predicting the occurrence of AD, we performed receiver operating characteristic (ROC) curve analyses using the pROC package of R (Robin et al., 2011). We selected the statistically significant genes from hub genes (*P* < 0.05) and used the nomogram to predict the occurrence of AD. The expression level of the hub genes was shown by the violin plot.

## Results

### Weighted Co-expression Network Construction and Identification of Core Modules

**FIGURE 2:**
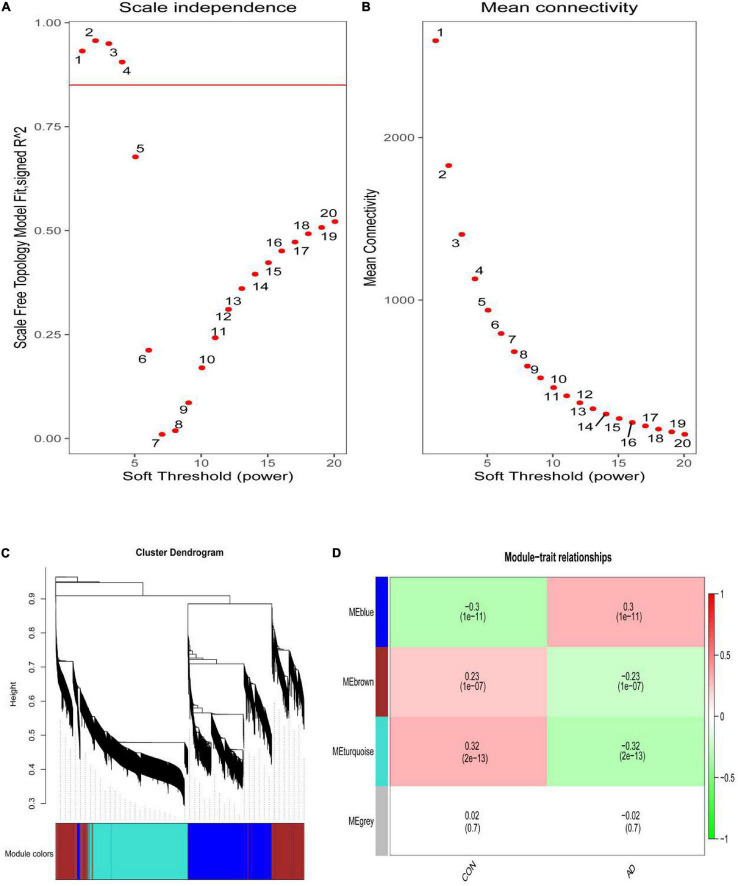
**(A)** Analysis of the scale-free index for various soft-threshold powers (β). **(B)** Analysis of the mean connectivity for various soft-threshold powers. **(C)** Identification of co-expression gene modules. The branches of the dendrogram cluster into 4 modules and each one was labeled in a unique color. **(D)** A heatmap showing the correlation between each module eigengene and phenotype. Two modules were correlated with AD-namely, turquoise and blue modules.

The scale-free network was constructed with the soft threshold set to 4 (R^2^ = 0.905) (Figures 2A,B). Then, the adjacency matrix and topological overlap matrix were built. We then calculated the module eigengenes representing the overall gene expression level of each module; these were clustered based on their correlation. A total of 4 modules were identified and labeled with a unique color (Figure 2C). We analyzed the correlations of each eigengene with phenotype (AD or control samples), and found two modules were correlated with AD-namely, the turquoise (cor = −0.32, *P* = 2e-13), and blue (cor = 0.30, *P* = 1e-11) modules (Figure 2D). The 4,617 genes in these modules-which are associated with AD-were retained for further analysis.

### The Extraction of Ferroptosis-Related Genes From FerrDb and Apoptosis-Related Genes From UniProt

The ferroptosis-related genes were downloaded and summarized from the FerrDb (Zhou and Bao, 2020; Table 1). 253 regulatory factors (including 108 drivers, 69 suppressors, 35 inducers, and 41 inhibitors), 111 markers, and 95 ferroptosis-related diseases were collated by FerrDb. We have extracted 2,130 genes from Uniprot, which is related to apoptosis.

**TABLE 1:** Details for FerrDb.

Data set	Category	Annotated from	Count	Annotations
Driver	Regulator	Gene	108	150
Suppressor	Regulator	Gene	69	109
Marker	Marker	Gene	111	123
Inducer	Regulator	Small molecule	35	54
Inhibitor	Regulator	Small molecule	41	46
Ferroptosis aggravates disease	Ferroptosis-disease association	Ferroptosis and disease	49	58
Ferroptosis alleviates disease	Ferroptosis-disease association	Ferroptosis and disease	46	77

*The number of “Count” and “Annotations” is inconsistent, because one gene can have multiple annotations.*

### Overlap Alzheimer’s Disease-Related Module Genes With Ferroptosis-Related Genes and Apoptosis-Related Genes, Respectively

**FIGURE 3:**
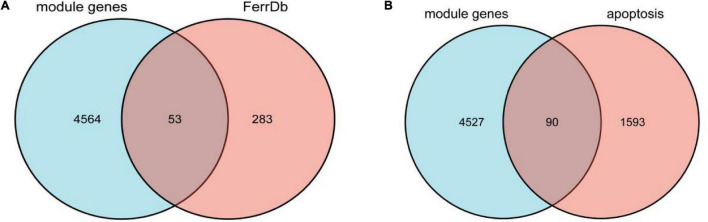
**(A)** Venn diagram showing the numbers of overlapped genes between AD-related module genes and ferroptosis-related genes. **(B)** Venn diagram showing the numbers of overlapped genes between AD-related module genes and apoptosis-related genes.

We overlapped the AD-related module genes derived from WGCNA with ferroptosis-related genes extracted from FerrDb, 53 overlapped genes were obtained, namely ferroptosis-related module genes, which was shown by the Venn diagram (Figure 3A). The details of overlapped genes, including 19 drivers, 16 suppressors, and 18 markers, were shown in Table 2. We also overlapped the AD-related module genes with apoptosis-related genes to obtain apoptosis-related module genes as a control for further analysis, and 90 overlapped genes were obtained, which was also shown by the Venn diagram (Figure 3B).

**TABLE 2:** Ferroptosis-related module genes obtained through the Venn diagram.

Type	Genes
Driver	PGD, YY1AP1, ATG3, ATG7, DPP4, NRAS, LPIN1, FBXW7, SCP2, EPAS1, TF, ATG16L1, IDH1, TFRC, BAP1, SNX4, PIK3CA, ATF3, PRKAA2
Suppressor	SQSTM1, SLC40A1, MTOR, FANCD2, MUC1, TP63, FTMT, PRDX6, NFE2L2, ACSL3, JUN, SLC7A11, FH, CISD2, SESN2, PROM2
Marker	TXNIP, HSD17B11, NCF2, PTGS2, ALB, STEAP3, SLC1A4, RRM2, CXCL2, ANGPTL7, PRDX1, SLC2A1, STMN1, RGS4, OXSR1, KLHL24, CAPG, DRD5

### Gene Ontology and Kyoto Encyclopedia of Genes and Genomes Enrichment Analysis of Overlapped Genes

**FIGURE 4:**
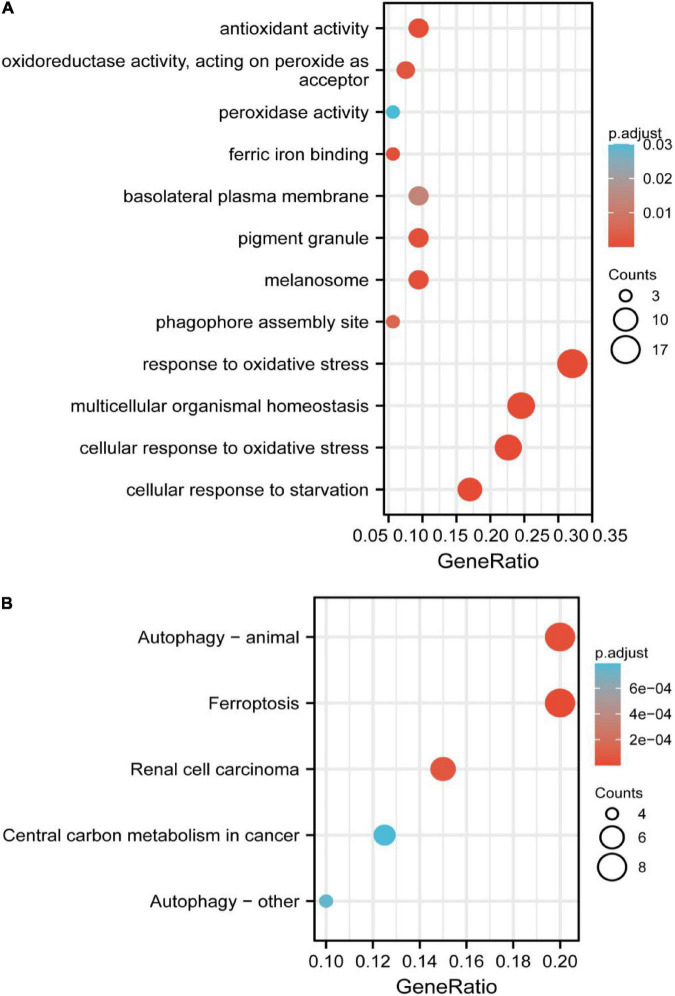
**(A)** Gene Ontology (GO) functional analysis showing enrichment of ferroptosis-related module genes. **(B)** Kyoto Encyclopedia of Genes and Genomes (KEGG) pathway enrichment analysis of ferroptosis-related module genes.

The significant GO functional terms of the 53 ferroptosis-related module genes, including BP, MF, and CC, were illustrated in Figure 4A. The significant terms of GO-BP were principally associated with the response to stress, such as the response to oxidative stress. The pathways enriched by GO-MF were principally associated with the activity of peroxidase, oxidoreductase, and antioxidant. The ferric iron-binding was also enriched by the GO-MF. The analysis of GO-CC indicated that overlapped genes were significantly enriched in basolateral plasma membrane, phagophore assembly site, pigment granule, and melanosome. The KEGG analysis showed that these overlapped genes were enriched not only in renal cell carcinoma pathways and central carbon metabolism in cancer, but also in autophagy-related pathways and ferroptosis (Figure 4B). The pathway of ferroptosis was enriched by KEGG, suggesting that these overlapped genes were significant for our study and could be used for further analysis.

### Protein-Protein Interaction Establishment and Identification of Hub Genes

**FIGURE 5:**
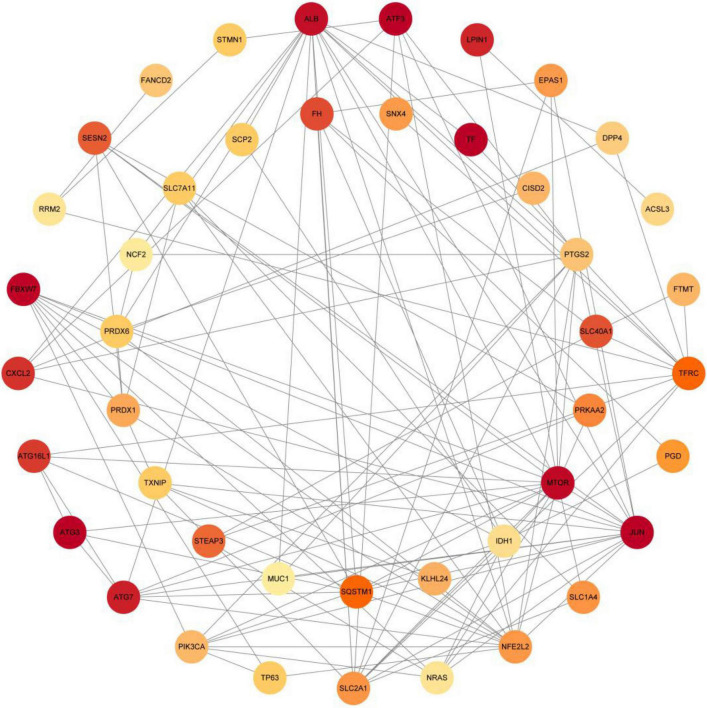
Protein-protein interaction network of 53 ferroptosis-related module genes were analyzed using Cytoscape software. The network includes 44 nodes and 120 edges (The disconnected nodes were hided). The edges between 2 nodes represent the gene-gene interactions. The size and color of the nodes corresponding to each gene were determined according to the degree of interaction. Color gradients represent the variation of the degrees of each gene from high to low.

**FIGURE 6:**
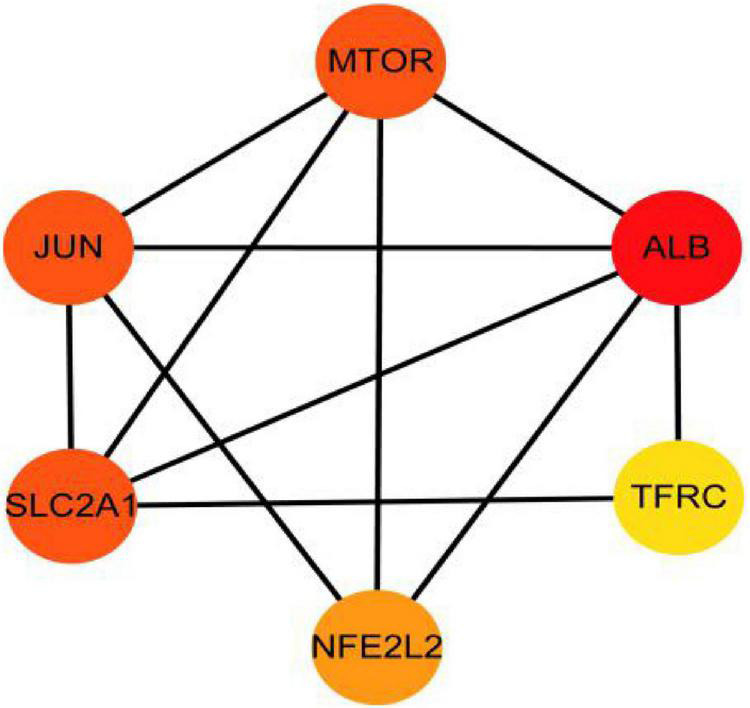
Protein–protein interaction network for the six hub genes. Three indicators (degree, closeness and betweenness) were, respectively, calculated to evaluate the importance of each node and the top 10 nodes were selected. The six hub genes were their common nodes.

The PPI analysis of 53 ferroptosis-related module genes was performed through the STRING database and visualized by Cytoscape V3.9.0 (Figure 5). JUN, SLC2A1, TFRC, ALB, MTOR, and NFE2L2 were taken as potential hub genes based on Degree, closeness, and betweenness. The hub genes were their common top ten nodes. The PPI network of the hub genes was presented in Figure 6. Similarly, the identification of hub genes of apoptosis-related module genes was also conducted, and STAT1, CFLAR, FASLG, MCL1 and BCL2L11 were obtained from the 90 overlapped genes.

### Construction and Validation of the Logistic Regression

**FIGURE 7:**
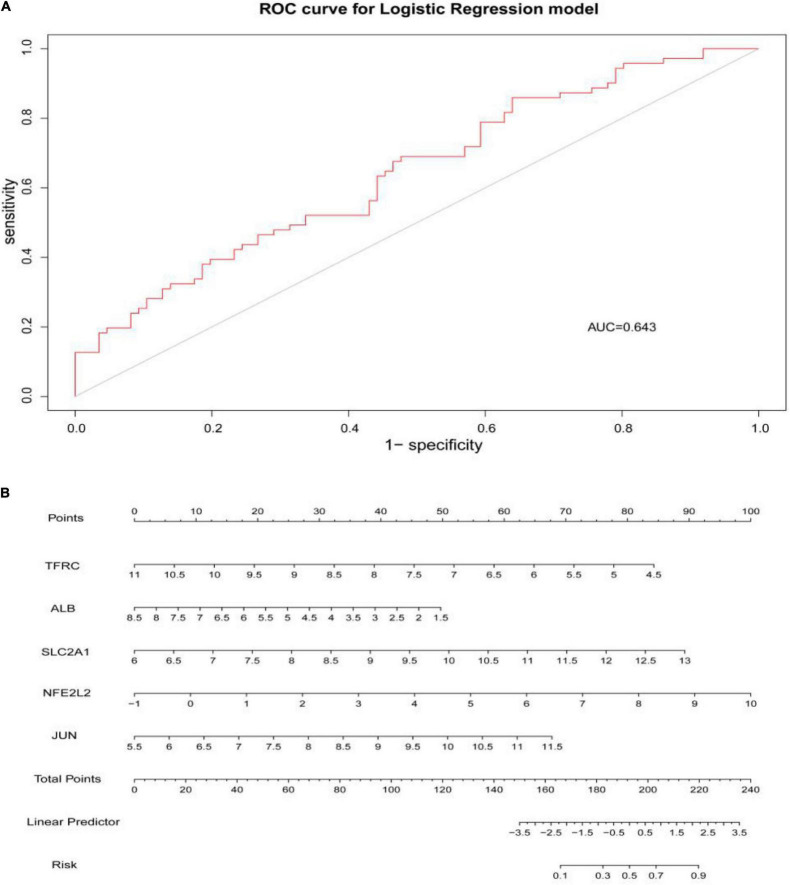
**(A)** ROC curve was used to evaluate the performance of the logistic regression model. The area under the curve (AUC) was 0.643. **(B)** The nomogram was used to predict the occurrence of AD. Ferroptosis-related hub genes, JUN, SLC2A1, TFRC, ALB, and NFE2L2 (P < 0.05), were included in this nomogram.

**FIGURE 8:**
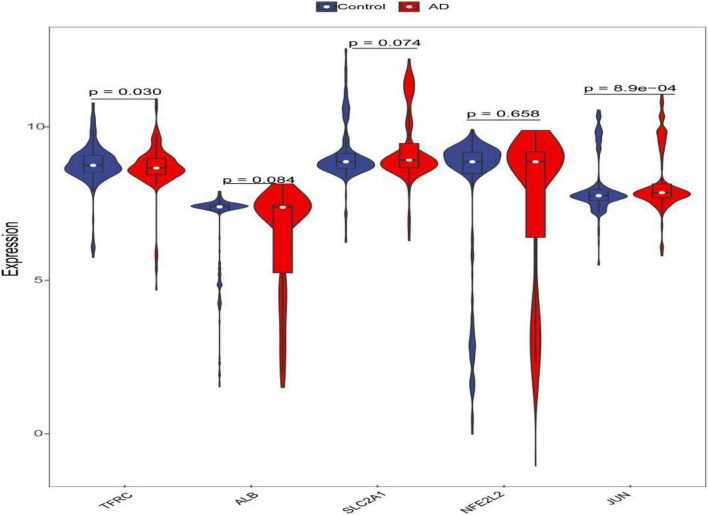
Violin plot of the expression level of five hub genes. The red violin reflects the AD group, and the blue violin reflects the control group.

**FIGURE 9:**
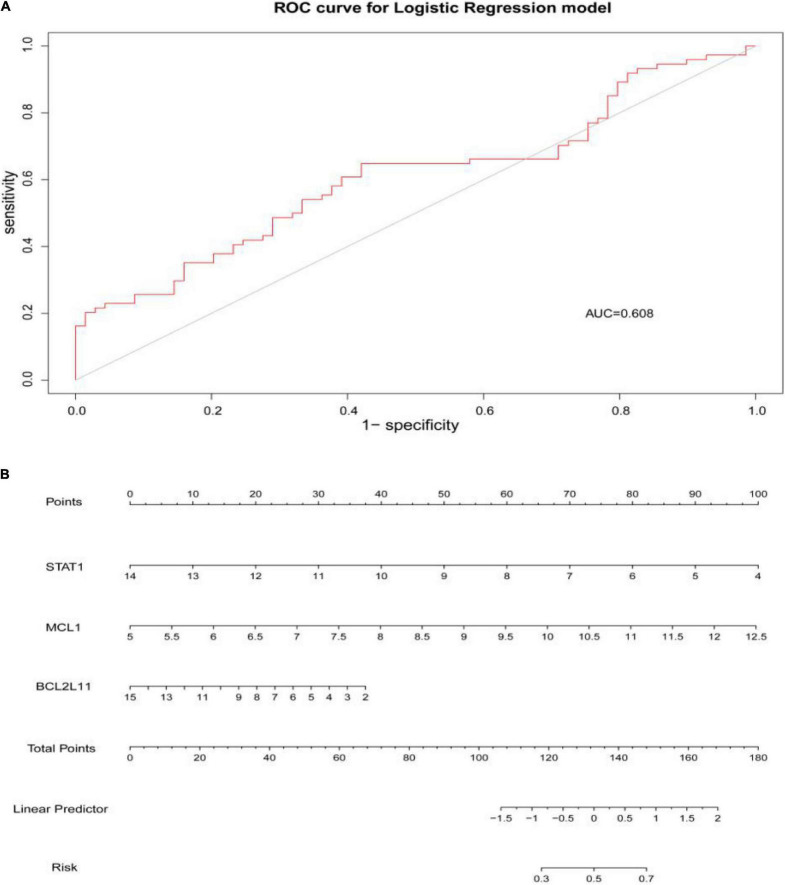
**(A)** The area under the curve (AUC) was 0.608. **(B)** The nomogram of apoptosis-related hub genes, STAT1, MCL1, and BCL2L11 (P < 0.05).

Through constructing the logistic regression, JUN, SLC2A1, TFRC, ALB, and NFE2L2 were selected, which could effectively differentiate AD patients from controls (*P* < 0.05). The P-value of MTOR was more than 0.05, which was not statistically significant. We used the ROC curve to evaluate the performance of the logistic regression model (the area under the ROC curve of the model was 0.643), and the nomogram was used for predicting the occurrence of AD (Figures 7A,B). The expression level of the five hub genes is shown in Figure 8. Similarly, the logistic regression was also constructed for apoptosis-related hub genes, and STAT1, MCL1, and BCL2L11 were selected and could distinguish AD patients from controls (P < 0.05). The AUC was 0.608, which was less than the former AUC value, suggesting that ferroptosis was more special than apoptosis in AD. The ROC curve and nomogram are shown in Figures 9A,B.

## Discussion

**FIGURE 10:**
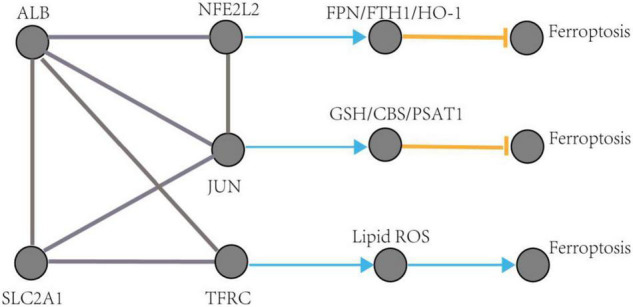
The regulation pathways of JUN, SLC2A1, TFRC, ALB, and NFE2L2 participating in ferroptosis in AD. The gray edge represents the gene-gene interactions. The orange T-shaped edge denotes suppression, and the blue arrow denotes promotion.

The pathological process of ferroptosis has some characteristics in common with AD, such as excess iron accumulation and elevated lipid peroxides. It has been reported that the pathological process of ferroptosis could be directly induced by iron overload (Wang et al., 2017; Fang et al., 2019). Clinically, lipid peroxidation metabolites were highly correlated with the progression of AD (Benseny-Cases et al., 2014). Besides, it has also been reported that reactive oxygen species (Wang et al., 2016) and reduced glutathione (Chiang et al., 2017) were found in the pathological process of AD. However, how does ferroptosis mediate AD? Some ferroptosis-related signaling pathways were found in AD, such as iron metabolism pathway, redox homeostasis pathway, and lipid metabolism pathway (Chen et al., 2021). Exploring of the mechanism of ferroptosis in AD could provide a novel therapeutic target for the treatment of AD and possibly, other neurodegenerative diseases (Ashraf and So, 2020). This study identified five hub genes that may participate in the pathologic processes associated with ferroptosis in AD. The possible pathways of these five genes involved in ferroptosis are shown in Figure 10 (see text footnote 2) (Gao et al., 2016; Shin et al., 2018; Chen et al., 2019).

Emerging evidence has demonstrated that ferroptosis could be a therapeutic target for AD (Gleason and Bush, 2021). Some ferroptosis inhibitors, such as iron-chelators and vitamin E, have shown clinical efficacy in treating AD. Deiprone is a brain osmotic iron-chelating agent currently in phase II clinical trials to treat AD (Nikseresht et al., 2019). Antioxidant vitamin E could delay decline in function and relieve caregiver burden in patients with AD (Dysken et al., 2014a,b). Collectively, Patients with AD may benefit from ferroptosis as a therapeutic target. Unlike targeting β-amyloid, the clinical trials of ferroptosis inhibitors are still in the exploratory stage and need to be dose-optimized and replicated on a larger scale (Nikseresht et al., 2019). The clinical efficacy of ferroptosis inhibitors in the treatment of AD also needs to be further improved.

There were some limitations to this study. Firstly, while selecting datasets for differentially expressed analysis, it was found that some datasets had fewer or no differentially expressed genes (DEGs, correcting *P*-value < 0.05 and | logFC| ≥ 1.0), such as GSE48350 (Berchtold et al., 2013) and GSE131617 (Miyashita et al., 2014; Kikuchi et al., 2020). Therefore, the datasets and related AD patients we can choose are still limited. In addition, if the DEGs further overlaps with the ferroptosis-related module genes, the number of available genes are limited and could not be used for further analysis. Secondly, the potential ferroptosis-related biomarkers identified by this study still need further literature support and laboratory evidence verification. Thirdly, the ferroptosis-related genes are derived from FerrDb, which is being updated continuously, and more genes are yet to be discovered.

## Conclusion

We identified five hub genes (JUN, SLC2A1, TFRC, ALB, and NFE2L2) that are closely associated with ferroptosis in AD and can differentiate AD patients from controls, and are thus potential ferroptosis-related biomarkers for disease diagnosis and therapeutic monitoring. Three hub genes of apoptosis-related genes in AD (STAT1, MCL1, and BCL2L11) were also identified as a control to show the specificity of Ferroptosis. JUN, SLC2A1, TFRC, ALB, and NFE2L2 are thus potential ferroptosis-related biomarkers for disease diagnosis and therapeutic monitoring.

## Data Availability Statement

The datasets presented in this study can be found in online repositories. The names of the repository/repositories and accession number(s) can be found in the article/supplementary material.

## Author Contributions

YW, GC, and WS contributed equally to this work. All authors contributed toward data analysis, drafted and critically revised the manuscript, gave final approval of the version to be published, and agreed to be accountable for all aspects of the work.

## Conflict of Interest

The authors declare that the research was conducted in the absence of any commercial or financial relationships that could be construed as a potential conflict of interest.

## Publisher’s Note

All claims expressed in this article are solely those of the authors and do not necessarily represent those of their affiliated organizations, or those of the publisher, the editors and the reviewers. Any product that may be evaluated in this article, or claim that may be made by its manufacturer, is not guaranteed or endorsed by the publisher.

## Footnotes

1
http://www.ncbi.nlm.nih.gov/geo

2
http://www.zhounan.org/ferrdb

3
https://www.uniprot.org/

4
https://string-db.org/

## References

[B1] Ashraf A. So P. W. (2020). Spotlight on Ferroptosis: iron-Dependent Cell Death in Alzheimer’s Disease. *Front. Aging Neurosci.* 12:196. 10.3389/fnagi.2020.00196 32760266 PMC7371849

[B2] Ayton S. Wang Y. Diouf I. Schneider J. A. Brockman J. Morris M. C. (2019). Brain iron is associated with accelerated cognitive decline in people with Alzheimer pathology. *Mol. Psychiatry* 25 2932–2941. 10.1038/s41380-019-0375-7 30778133 PMC6698435

[B3] Benseny-Cases N. Klementieva O. Cotte M. Ferrer I. Cladera J. (2014). Microspectroscopy (muFTIR) reveals co-localization of lipid oxidation and amyloid plaques in human Alzheimer disease brains. *Anal. Chem.* 86 12047–12054. 10.1021/ac502667b 25415602

[B4] Berchtold N. C. Coleman P. D. Cribbs D. H. Rogers J. Gillen D. L. Cotman C. W. (2013). Synaptic genes are extensively downregulated across multiple brain regions in normal human aging and Alzheimer’s disease. *Neurobiol. Aging* 34 1653–1661. 10.1016/j.neurobiolaging.2012.11.024 23273601 PMC4022280

[B5] Chen K. Jiang X. Wu M. Cao X. Bao W. Zhu L. Q. (2021). Ferroptosis, a Potential Therapeutic Target in Alzheimer’s Disease. *Front. Cell Dev. Biol.* 9:704298. 10.3389/fcell.2021.704298 34422824 PMC8374166

[B6] Chen Y. Zhu G. Liu Y. Wu Q. Zhang X. Bian Z. (2019). O-GlcNAcylated c-Jun antagonizes ferroptosis via inhibiting GSH synthesis in liver cancer. *Cell Signal* 63:109384. 10.1016/j.cellsig.2019.109384 31394193

[B7] Chiang G. C. Mao X. Kang G. Chang E. Pandya S. Vallabhajosula S. (2017). Relationships among cortical glutathione levels, brain amyloidosis, and memory in healthy older adults investigated *in vivo* with H-1-MRS and pittsburgh compound-B PET. *Am. J. Neuroradiol.* 38 1130–1137. 10.3174/ajnr.a5143 28341718 PMC5471116

[B8] Dysken M. W. Guarino P. D. Vertrees J. E. Asthana S. Sano M. Llorente M. (2014a). Vitamin E and memantine in Alzheimer’s disease: clinical trial methods and baseline data. *Alzheimer’s Dement.* 10 36–44. 10.1016/j.jalz.2013.01.014 23583234 PMC4128187

[B9] Dysken M. W. Sano M. Asthana S. Vertrees J. E. Pallaki M. Llorente M. (2014b). Effect of vitamin E and memantine on functional decline in Alzheimer disease: the TEAM-AD VA cooperative randomized trial. *JAMA* 311 1161–1161. 10.1001/jama.2013.282834 24381967 PMC4109898

[B10] Fang X. X. Wang H. Han D. Xie E. J. Yang X. Wei J. Y. (2019). Ferroptosis as a target for protection against cardiomyopathy. *Proc. Natl. Acad. Sci. U S A.* 116 2672–2680. 10.1073/pnas.1821022116 30692261 PMC6377499

[B11] Gao M. Monian P. Pan Q. Zhang W. Xiang J. Jiang X. (2016). Ferroptosis is an autophagic cell death process. *Cell Res.* 26 1021–1032. 10.1038/cr.2016.95 27514700 PMC5034113

[B12] Gbd 2016 Dementia Collaborators. (2019). Global, regional, and national burden of Alzheimer’s disease and other dementias, 1990-2016: a systematic analysis for the Global Burden of Disease Study 2016. *Lancet Neurol.* 18 88–106. 10.1016/S1474-4422(18)30403-4 30497964 PMC6291454

[B13] Gleason A. Bush A. I. (2021). Iron and Ferroptosis as Therapeutic Targets in Alzheimer’s Disease. *Neurotherapeutics* 18 252–264. 10.1007/sl3311-020-00954-y 33111259 PMC8116360

[B14] Hambright W. S. Fonseca R. S. Chen L. Na R. Ran Q. (2017). Ablation of ferroptosis regulator glutathione peroxidase 4 in forebrain neurons promotes cognitive impairment and neurodegeneration. *Redox Biol.* 12 8–17. 10.1016/j.redox.2017.01.021 28212525 PMC5312549

[B15] Jakaria M. Bush A. I. Ayton S. (2021). Ferroptosis as a mechanism of neurodegeneration in Alzheimer’s disease. *J. Neurochem.* 159 804–825. 10.1111/jnc.15519 34553778

[B16] Kikuchi M. Sekiya M. Hara N. Miyashita A. Kuwano R. Ikeuchi T. (2020). Disruption of a RAC1-centred network is associated with Alzheimer’s disease pathology and causes age-dependent neurodegeneration. *Hum. Mol. Genet.* 29 817–833. 10.1093/hmg/ddz320 31942999 PMC7191305

[B17] Lane D. J. R. Ayton S. Bush A. I. (2018). Iron and Alzheimer’s Disease: an Update on Emerging Mechanisms. *J. Alzheimers Dis.* 64 S379–S395. 10.3233/JAD-179944 29865061

[B18] Langfelder P. Horvath S. (2008). WGCNA: an R package for weighted correlation network analysis. *BMC Bioinformatics* 9:559. 10.1186/1471-2105-559 PMC2631488 19114008

[B19] Langfelder P. Horvath S. (2012). Fast R Functions for Robust Correlations and Hierarchical Clustering. *J. Statist. Softw.* 46:11. PMC3465711 23050260

[B20] Masaldan S. Bush A. I. Devos D. Rolland A. S. Moreau C. (2019). Striking while the iron is hot: Iron metabolism and ferroptosis in neurodegeneration. *Free Radic. Biol. Med.* 133 221–233. 10.1016/j.freeradbiomed.2018.09.033 30266679

[B21] Mckay E. C. Beck J. S. Khoo S. K. Dykema K. J. Cottingham S. L. Winn M. E. (2019). Peri-Infarct Upregulation of the Oxytocin Receptor in Vascular Dementia. *J. Neuropathol. Exp. Neurol.* 78 436–452. 10.1093/jnen/nlz023 30990880 PMC6467199

[B22] Miyashita A. Hatsuta H. Kikuchi M. Nakaya A. Saito Y. Tsukie T. (2014). Genes associated with the progression of neurofibrillary tangles in Alzheimer’s disease. *Transl. Psychiatry* 4:e396. 10.1038/tp.2014.35 26126179 PMC4080317

[B23] Nikseresht S. Bush A. I. Ayton S. (2019). Treating Alzheimer’s disease by targeting iron. *Br. J. Pharmacol.* 176 3622–3635. 10.1111/bph.14567 30632143 PMC6715619

[B24] Obulesu M. Lakshmi M. J. (2014). Apoptosis in Alzheimer’s disease: an understanding of the physiology, pathology and therapeutic avenues. *Neurochem. Res.* 39 2301–2312. 10.1007/s11064-014-1454-4 25322820

[B25] Plascencia-Villa G. Perry G. (2021). Preventive and Therapeutic Strategies in Alzheimer’s Disease: focus on Oxidative Stress. *Antioxid. Redox Signal.* 34 591–610. 10.1089/ars.2020.8134 32486897 PMC8098758

[B26] Robin X. Turck N. Hainard A. Tiberti N. Lisacek F. Sanchez J. C. (2011). pROC: an open-source package for R and S + to analyze and compare ROC curves. *BMC Bioinform.* 12:77. 10.1186/1471-2105-12-77 21414208 PMC3068975

[B27] Shannon P. Markiel A. Ozier O. Baliga N. S. Wang J. T. Ramage D. (2003). Cytoscape: a software environment for integrated models of biomolecular interaction networks. *Genome Res.* 13 2498–2504. 10.1101/gr.1239303 14597658 PMC403769

[B28] Sharma V. K. Singh T. G. Singh S. Garg N. Dhiman S. (2021). Apoptotic Pathways and Alzheimer’s Disease: probing Therapeutic Potential. *Neurochem. Res.* 46 3103–3122. 10.1007/s11064-021-03418-7 34386919

[B29] Shimohama S. (2000). Apoptosis in Alzheimer’s disease–an update. *Apoptosis* 5 9–16. 10.1023/a:1009625323388 11227497

[B30] Shin D. Kim E. H. Lee J. Roh J. L. (2018). Nrf2 inhibition reverses resistance to GPX4 inhibitor-induced ferroptosis in head and neck cancer. *Free Radi. Biol. Med.* 129 454–462. 10.1016/j.freeradbiomed.20180.10.426 30339884

[B31] Tan M. G. Chua W. T. Esiri M. M. Smith A. D. Vinters H. V. Lai M. K. (2010). Genome wide profiling of altered gene expression in the neocortex of Alzheimer’s disease. *J. Neurosci. Res.* 88 1157–1169. 10.1002/jnr.22290 19937809

[B32] Vitalakumar D. Sharma A. Flora S. J. S. (2021). Ferroptosis: a potential therapeutic target for neurodegenerative diseases. *J. Biochem. Mol. Toxicol.* 35:e22830. 10.1002/jbt.22830 34047408

[B33] Wang H. An P. Xie E. Wu Q. Fang X. Gao H. (2017). Characterization of ferroptosis in murine models of hemochromatosis. *Hepatology* 66 449–465. 10.1002/hep.29117 28195347 PMC5573904

[B34] Wang X. Hu X. Yang Y. Takata T. Sakurai T. (2016). Nicotinamide mononucleotide protects against β-amyloid oligomer-induced cognitive impairment and neuronal death. *Brain Res.* 1643 1–9. 10.1016/j.brainres.2016.04.060 27130898

[B35] Weiland A. Wang Y. Wu W. Lan X. Han X. Li Q. (2019). Ferroptosis and Its Role in Diverse Brain Diseases. *Mol. Neurobiol.* 56 4880–4893. 10.1007/s12035-018-1403-3 30406908 PMC6506411

[B36] Yan N. Zhang J. (2019). Iron Metabolism. *Front. Neurosci.* 13:1443. 10.3389/fnins.2019.01443 32063824 PMC7000453

[B37] Yu G. Wang L. G. Han Y. He Q. Y. (2012). clusterProfiler: an R package for comparing biological themes among gene clusters. *OMICS* 16 284–287. 10.1089/omi.2011.0118 22455463 PMC3339379

[B38] Zhang C. Rodriguez C. Spaulding J. Aw T. Y. Feng J. (2012). Age-dependent and tissue-related glutathione redox status in a mouse model of Alzheimer’s disease. *J. Alzheimers Dis.* 28 655–666. 10.3233/JAD-2011-111244 22045490 PMC4221633

[B39] Zhang G. Zhang Y. Shen Y. Wang Y. Zhao M. Sun L. (2021). The Potential Role of Ferroptosis in Alzheimer’s Disease. *J. Alzheimers Dis.* 80 907–925. 10.3233/JAD-201369 33646161

[B40] Zhou N. Bao J. (2020). FerrDb: a manually curated resource for regulators and markers of ferroptosis and ferroptosis-disease associations. *Database J. Biol. Databases Curation* 2020:baaa021. 10.1093/database/baaa021 32219413 PMC7100629

